# Analysis of Opioid Poisoning in Medically Underserved Rural Areas: An Evaluation of International Statistical Classification of Diseases Codes from the State of South Dakota

**Published:** 2022-10-14

**Authors:** Ahmed Nahian, Jewel Goodman Shepherd

**Affiliations:** 1 Center of Brain and Behavior Research, University of South Dakota, 901 Rose St. Apt 133-A, Vermillion, SD 57069, USA; 2 University of South Dakota, 414 E. Clark St. Vermillion, SD 57069, USA

**Keywords:** Addiction, Addiction research, Addiction therapy, Rural, Underserved, Addiction, Opioid, Heroin, Federal (MeSH/MEDLINE)

## Abstract

**Background:**

Rural hospitals and patient population tend to be medically underserved. The states with more rural population dispensed the most opioids per person in the last 10 years. We aimed to explore if rurality contributed to the likelihood of higher opioid adversity and how it affected substance-use rehabilitation in federally designated Medically Underserved Areas (MUAs).

**Methods:**

We analyzed data dispensed by the South Dakota Department of Health (DOH) on opioid-led poisoning International Classification of Disease (ICD) codes that were active within the state in the last decade. After locating MUA rural and partially rural counties, we cross profiled the counties to the state datasets. Assessments were conducted using the PROC SURVEY methods in SAS version 9.3 (SAS Institute) and checked for multicollinearity with the Belsley-Kuh-Welsch technique. Finally, we used the American Hospital Association (AHA) database for analyzing substance use rehabilitation availability on per hospital basis.

**Results:**

The chi-square statistic for comparing opioid codes against non-opioid codes distributed among three categories, rural, non-rural, and partially rural was significant at the limit of p <0.05. 81.134% of opioid-led poisoning codes were activated in a rural county. Only four hospitals had substance-use rehabilitation, three of which were in a non-rural area. More people from the teenage and early-adulthood years (10–19) were prone to opioid usage.

**Conclusions:**

Rural counties in South Dakota were more likely to dispense opioid care and not have access to rehabilitation. We also found that as the opioid dispensing rate at hospitals within a state decreased as the state had less rural counties. Introducing public programs to train more physicians and cutting down cost of non-opioid based care may lower opioid distribution and increase rehabilitation options in rural hospitals.

## Introduction

Despite federal guidelines that highlight prescription opioids are not advised as a first-line medication for most forms of pain due to the substantial dangers of opioid use disorders (OUDs) and overdose, they have been widely utilized to treat both chronic and acute pain in the United States. Analyzing the trends of prescriptive opioid usage can assist to strategies to encourage safer and more potent pain management, as OUD-led deaths remain a public health crisis. The United States is contending with an ongoing opioid crisis, whereby in 2018, opioids participated in two out of three drug-related deaths [1]. The Department of Health and Human Services (HHS) declared a public health emergency and unveiled a 5-Point Strategy to Combat the Opioid Crisis in 2017 [1]. About 8.7% of non-elderly individuals completed at least one outpatient opioid script, with 2.3% filling five or more prescriptions in the period between 2018 and 2019 [2].

There are currently 6093 U.S. hospitals, 5139 of which are classified as rural community hospitals by the U.S. Office of Management and Budget [3]. Although non-hospital-dispensed prescription opioid morbidity and mortality have increased in all states over the last decade, injury and death from nonmedical prescription opioid usage are concentrated in states with substantial rural areas, such as Alaska, Montana, Colorado, and North Dakota [4]. The said states topped hospital opioid distribution per person by weight in 2019 [2]. These numbers have altered little since 2012, when South Carolina, Alaska, North Dakota, and South Dakota had the largest dispersion. Alaska and North Dakota continue to have the greatest weight-per-person opioid deliveries in hospitals [4].

The distinctions between urban and rural locations are not simple, but rather represent a spectrum of population density and accessibility to the United States’ 1098 defined metropolitan locations [5]. South Dakota is 75,885 square miles in size, with a projected population of 886,667 people in 2020, with 445,734 people residing in rural areas [5]. With only 65 federally qualified health centers, South Dakota tends to be a medically underserved state. As researchers from one of the few research universities in the state, we were interested in zooming into every county within South Dakota and explore if rurality played a direct role in the fate of a hospital becoming a frequent distributor for opioids. We designed our project to determine if rural hospitals within a state encounter more opioid poisoning cases than hospitals located in its non-rural areas and the likelihood of a hospital to provide substance-use rehabilitation based on its rurality. Using the “MUA Find” tool from the Health Resources & Services Administration (HRSA), we located every rural county within our state.

This project also utilized the most current national trends to explore the factors associated with patient ED encounters specific to ICD codes related to substance use. We had set main parameters of analysis once we knew what counties are rural vs. non-rural: 1) How often are opioid overdoses occurring within a county 2) Are the non-opioid services Centers for Medicare & Medicaid Services (CMS)-encoded/ covered by Medicaid/Medicare? We hypothesized that healthcare facilities in rural areas are likely to encounter more opioid cases than non-opioid cases; we also hypothesized that hospitals in non-rural areas are likely to offer more substance-use rehabilitation services than hospitals in rural areas.

## Methods

### Study design and sample

This study examined the DOHSD -released reports of opioid-led poisoning International Classification of Disease (ICD) codes that were active within randomized counties from 2016 to 2020. South Dakota has been one of the top 10 states to distribute opioids from hospital facilities for over ten years [4]. Only data that is within ICD10CMF’s T36-T65 codes in counties with a defined rural status by the HRSA were included. Counties were excluded if they had an “unknown” rural status, resulting in non-equivalent groups. We then conducted a survey of service availability for substance-use rehabilitation using the AHA database.

### Variables of interest

The database from the State Department of the State of South Dakota organized the ED codes by counties. The database consisted of de-identified patients who visited an ED within one of the counties in South Dakota with a poisoning listed in ICD10CMF’s T36-T65. The *International Classification of Diseases, Ninth Revision, Clinical Modification* (ICD-9-CM) system is the recognized clinical system for diagnosing patients and submitting claims for insurance payment.

Among all codes, if there was an indication of T39, we omitted that code for the purpose of this study since it is classified as “Poisoning by, adverse effect of and underdosing of nonopioid analgesics, antipyretics and antirheumatics” [6]. The counties listed in the database must also be in the “MUA Find” application in the HRSA database and have a functional rural status to be included in the study. If it did not exist in the database or has an “Unknown” rural status, we omitted the code. Interventions not labeled “Unknown” by HRSA but having a “Partially Rural” status could match the ≥4 National Association of Social Workers (NASW) criteria, which is why we included it in the meta-analysis. An MUA is defined by federal guidelines, which derives from calculating an index of medical underservice (IMU) since 1975. We decided to include rurality listings even if they displayed old listings since HRSA audits and updates their system on a regular basis and an area could hold the same status of rurality for undesignated number of years [7]. Opioid poisoning (OP) may include several types or brands of opioid such as synthetic, methadone, fentanyl, etc., but refers to the drug-led poisoning relating to opium.

The primary reason for ED visit was identified as the first listed poisoning ICD code among the possible 29 reported ICD codes regarding substance use-led poisoning. There were 891 T39 codes with non-opioid designation utilized to ascertain the reason for visit; because it does not fall within the parameter of our analysis, we omitted any result that the software pinpointed with the code. Discharge status was also examined; the discharge categories associated with each visit were: discharged (patient who received treatment and left the ED with discharge), admitted (patient admitted to the institution holding the ED), or other (expired in the ED, transferred, or left against medical advice). Other patient demographic traits available in dataset and assessed in this study were age, sex, race, and ethnicity. The median household income of the county of ICD activation along with other socioeconomic listings were extracted from Data USA’s profiles, which are based on reports from federal agencies and is an ongoing partnership between Deloitte, Massachusetts Institute of Technology Collective Learning Group, and Datawheel.

We also used the AHA Database to profile all hospitals located in South Dakota to map out the promptness of care. Medicare Severity Diagnosis Related Groups (MS-DRGs) are grouped by “Medical Service” to provide a profound method of summarizing utilization data. We aimed to look if MS-DRGs 894, 895, 896, and 897 were offered in a caregiving facility, because they represent rehabilitation of substance dependence.

### Data analysis

To compare the ED visits, we separated reason of visit by OP-related and non-OP-related categories and used χ^2^ tests in Statistical Analysis System (SAS) for comparing it to geographic characteristics. Among OP-related ED visits, the proportions of visits by rurality status for specific poisoning types and for each of the top primary opioid code for ED visit were estimated. Furthermore, among the OP-related ED visits only, a weighted multivariable logistic regression model was used to examine demographic and clinical factors associated with inpatient admission. Assessments were conducted using the PROC SURVEY methods in SAS version 9.3 (SAS Institute) and SUDAAN 11.0.0 (Research Triangle Institute).

Data were checked for multicollinearity with the Belsley-Kuh-Welsch technique. Heteroskedasticity and normality of residuals were assessed respectively by the White test and the Lilliefors test.

We also made a profile for all counties within South Dakota based off their rurality status and compared it to their respective opioid dispensing rate by 100 as reported by CDC. Numeric variables were expressed as mean (±SD) and discrete outcomes as absolute and relative (%) frequencies. We created 2 groups according to the values of Opioid Dispensing Rate per 100. Group comparability was assessed by comparing baseline demographic data and follow-up duration between groups. Normality and heteroskedasticity of continuous data were assessed with Shapiro-Wilk and Levene’s test, respectively. Continuous outcomes were compared with unpaired Student t-test, Welch t-test or Mann-Whitney U test according to data distribution. Discrete outcomes were compared with chi-squared or Fisher’s exact test accordingly. The alpha risk was set to 5% and two-tailed tests were used.

To correlate availability of substance abuse rehabilitation service to geographic location, we used Pearson’s *r*. We compared the Pearson’s *r* to our χ^2^ test results for further mapping out the comparison to rurality status.

## Results

### Characteristics of the study sample

**Figure 1:**
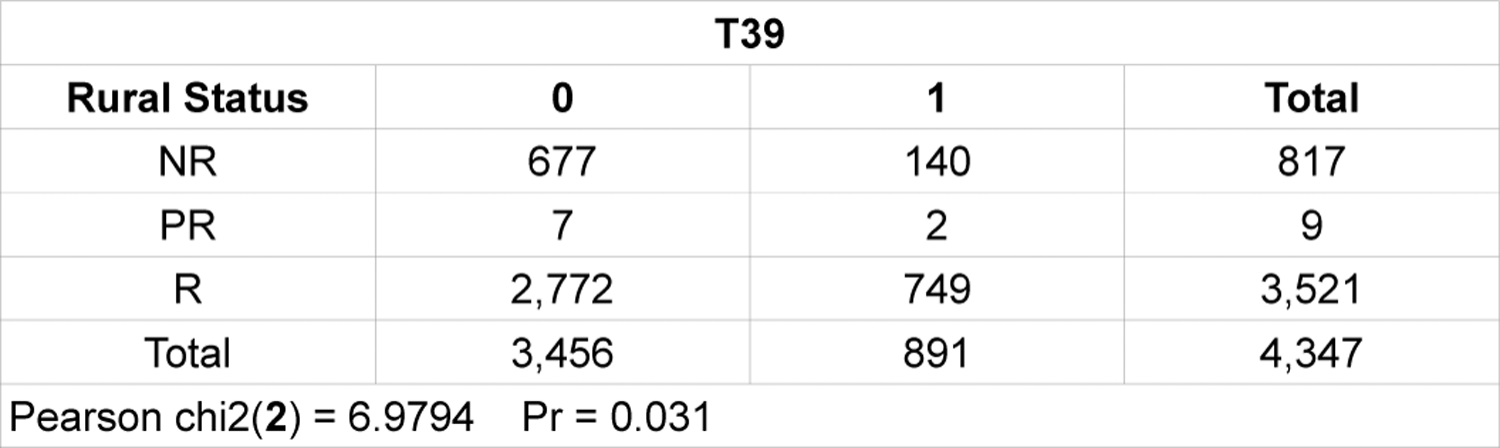
SAS Chi-Square Test for OP v. Non-OP Based on Rurality.

**Figure 2:**
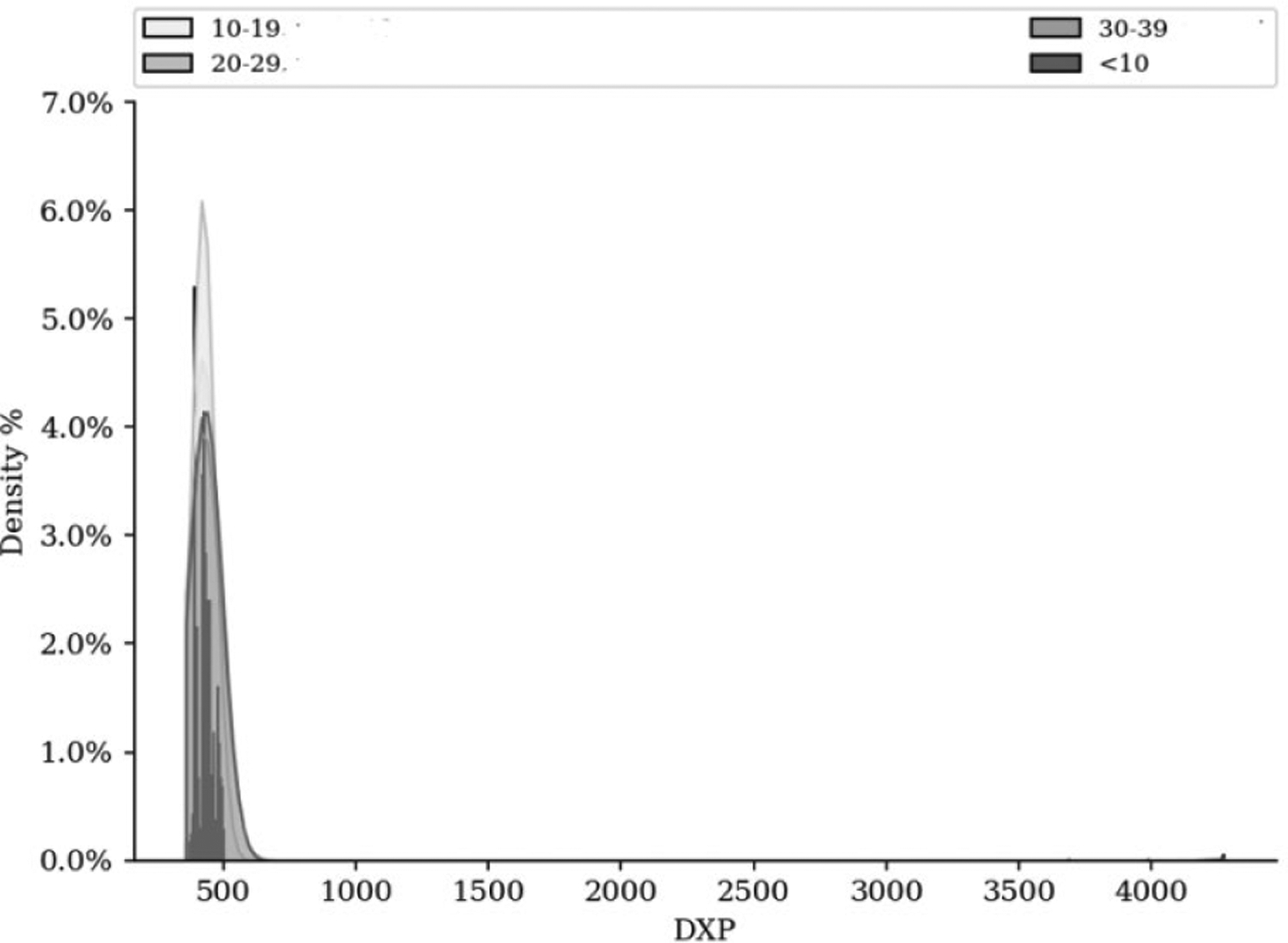
Bivariate Distribution of Age.

The DOHSD-dispensed data included a total of 4,347 county-based ICD codes admitted to an ED in the range of 2016 to 2020. Of these codes, 0% were excluded based on first exclusion criteria because 100% of the counties had a designated rural status at the MUA locator (a summary search engine made by the HRSA), leaving 4,347 codes for analysis; however, we eliminated 891 results from the final analysis because they were T39 codes, which codes for nonopioid substance poisoning. The secondary exclusion criteria resulted in a cohort of 3,456 codes. We individually analyzed each of the three categories to select for and omit non-opioid codes. All codes came from the same ICD listing and were within a county in South Dakota. In our initial cohort, 80.998% of the codes were activated in a rural county, 0.207% of codes were within a partially rural county; after the final cohort, we figured that 81.134% of the codes were activated in a rural county while 0.174% of codes came from an ED in a partially rural area. The percentage of patients who had an opioid poisoning within a nun-rural county was 18.795% in the initial cohort, which became 18.692% in the final cohort. The differences between initial and final cohorts for rural, partially rural, and non-rural areas were 0.545%, 16.146%, and 0.168%, respectively (Figures 1 and 2).

### Opioid poisoning v. Non-opioid poisoning

We conducted univariate and multivariate logistic regression to interpret associations between various opioid poisoning codes (all codes within ICD T36-&65, except the T39 codes) and rurality. We abbreviated the ICD codes by the first three digits for convenience of regression. For univariate regression, the Kruskal test was used to compare the principal diagnosis (DXP) median according to Rural Status. The Mann-Whitney test was used to compare the DXP median according to Rural Status in multivariate regression. The alpha risk was set to 0.05. We conducted both univariate and multivariate regression analyses. The median DXP for patients were respectively 428.0 (Q1 393.0; Q3 436.0), 426.0 (Q1 401.0; Q3 436.0) and 393.0 (Q1 393.0; Q3 393.0) for patients with Rural Status= R (reference), NR and PR (p <0.001). In multivariate analysis, DXP (OR=0.97, [0.95; 0.98], p 0.0001) were associated with lower rates of Rural Status.

Results of the multivariable χ^2^ examining the associations between opioids and rurality of the location of hospital among the poisoning-related visits are shown on Table 1 (Table 1). The chi-square statistic for comparing opioid codes against non-opioid codes distributed among three categories, rural, non-rural, and partially rural was 6.9794. The p-value was .031. The result was significant at p < .05.

### Opioid dispensing

The proportion of groups NR, PR and R across the profile of cumulative counties within South Dakota were respectively 9.09%, 3.03% and 87.88% in patients for which Opioid Dispensing Rate per 100>= 19.8 was Yes and 0.0%, 3.12% and 96.88% in patients for which Opioid Dispensing Rate per 100>= 19.8 was No (p=0.238).

### Substance use rehabilitation services in rural areas and miscellaneous trends

Out of 65 hospitals, only four offered substance-use rehabilitation; three out of the four hospitals were in a non-rural area. Demographic variables significantly associated with increased odds of admission were increasing age. The age range of 10–19 had the highest count of OP-led poisoning (N=1429) compared to any other age-based cohort. Females (N=2672) had statistically significant decreased odds of encountering an OP-led poisoning compared with males (N=1675). Patients identifying as “White” were more likely to have an OP-led poisoning event than another cohort.

In multivariate analysis, DXP/ICD codes were related with Ethnicity = 3 (β=−18.17, [−86.34; 49.99], p= 0.6012), Ethnicity = 1 (β=−7.33, [−40.89; 26.23], p= 0.6684), Age Group = 20–29 (β=−5.34, [−21.11; 10.42], p= 0.5064), Ethnicity = 4 (β=−1.97, [−26.05; 22.11], p= 0.8726), Age Group = 30–39 (β=2.28, [−15.73; 20.3], p= 0.8038), Age Group = <10 (β=5.0, [−14.5; 24.5], p= 0.615). We also conducted a univariate analysis of miscellaneous trends. The median DXP for patients were respectively 432.0 (Q1 393.0; Q3 436.0), 426.0 (Q1 401.0; Q3 436.0), 426.0 (Q1 402.0; Q3 435.0) and 432.0 (Q1 393.0; Q3 454.0) for patients with Age Group= 10–19 (reference), 20–29, 30–39 and <10 (p <0.001). The median DXP for patients were respectively 432.0 (Q1 393.0; Q3 436.0), 430.0 (Q1 393.0; Q3 435.0), 432.0 (Q1 393.0; Q3 450.0) and 414.0 (Q1 393.0; Q3 434.25) for patients with Ethnicity= 2 (reference), 4, 1 and 3 (p=0.051).

## Discussion

Opioids are the commonly accepted and used form of analgesic against pain rehabilitation. Through the results of this study, we observed that most ICD codes about substance-led poisoning related to opioid in the State of South Dakota. As stated earlier, we omitted 891 results from the final analysis because they were T39 codes, which indicate primary diagnoses of poisoning led by non-opioid substance. As a result, the differences between initial and final groups for rural, partially rural, and non-rural areas were 0.545%, 16.146%, and 0.168%, respectively. With exception to partially rural cohort (N=9), all other differences remained miniscule, indicating that most substance-led poisoning in the state happened because of opioids. It is important to note that the partially rural group is so small that it fails to compare as significantly to the other groups in the study. When the opiate pathway is excessively and unopposed stimulated, opioid overdose develops. This may result in diminished breathing effort and death. Overdoses using opioids are happening more frequently. Opioids are the most widely used drugs, and drug overdose is the major cause of sudden death in the United States. The CDC presently estimates that every day, opioid usage results in more than 1000 visits to emergency rooms and about 91 overdose deaths [8].

One important concept is that there are multiple categories of opioids that cause poisoning. While not all type of opioids is prescribed as medications, they still fall within the category of such poisoning. Misuse of such opioids often tend to reflect on an aftereffect of medically prescribed opioids and affordability of the relief. Heroin is up to ten times more affordable and accessible than prescription opioid drugs sold on the street, which typically cost a dollar per milligram; a bag of heroin costs about $2 [9]. Furthermore, there is a growing trend of fentanyl and other synthetic opioid compounds getting added to heroin. As a result, there is a greater chance of overdosing and varying opioid potency proportions [9]. The bulk of illegal narcotics sold on the street are frequently tainted with additional drugs. Without the end user’s knowledge, dealers frequently add additional agents to the recipe to boost earnings. These additives frequently have pharmacological activity. Heroin had been tainted with scopolamine in New York 20 years prior, causing serious anticholinergic poisoning; there is also a current surge of cocaine adulteration [9, 10].

Furthermore, we noticed that most codes were activated in a hospital located within an HRSA designated MUA rural area. The HRSA defines “rural” counties by following the Rural-Urban Commuting Area (RUCA) codes: a) All non-metro counties; b) All metro census tracts coded RUCA 4–10; c) Large area Metro census tracts of at least 400 sq. miles in area with population density of 35 or less per sq. mile with RUCA codes 2–3; d) all outlying metro counties without a UA (from Fiscal Year 2022 Rural Health Grants) [11]. The HRSA does not strictly adhere to the United States Census Bureau (Census) and the Office of Management and Budget (OMB) definitions and designation of counties and rurality. The reference their definitions and hybridizes with Rural-Urban Commuting Area (RUCA) codes to compose their own designations. According to data dispensed by the HRSA, there are 76 counties eligible for HRSA rural designation within South Dakota. Most counties within South Dakota are considered “rural” by the HRSA (N=70); only 2 counties are designated as “partially rural”, and 4 counties are designated as “non-rural.” In such situation, it is natural to question the validity of correlating the frequency of codes to the geographic location since most counties are rural; there may have been more codes in rural areas because there are comparatively more rural areas. The statistics, however, narrated a different story.

We statistically associated HRSA rural status to types of ICD codes activated from the ED by first conducting a T-Test on SAS. We wanted to explain the DXP considering the rurality status, therefore. As shown earlier, the result was significant at an acceptable margin of p<0.05. We further evaluated this association using Kruskal test. DXP is a continuous numerical variable compared across the modalities of Rural Status. We, therefore, relied with the first run of results and associated the likelihood of an opium-led poisoning by the rurality status.

With rurality as an established factor of significance, it was important for us to consider every county within South Dakota, one of the Top 5 states to dispense the most opioids (currently number 6) within the last two decades. Compared to states with more metropolitan areas, states with more medically underserved areas (MUAs) with rural designation dispensed the most opioids per person in the last 10 years [12, 13]. The association between Opioid Dispensing Rate per 100>= 19.8, the median of dispensing rates, and Rurality Status was assessed with the Fisher’s exact test. The alpha risk was set to 0.05. All cells of the contingency table have an expected count of at least 1, but less than 80% of the cells of the contingency table had an expected count of at least 5. Therefore, according to Yates, Moore and & McCabe, a Chi-squared test was not feasible, and a Fisher’s exact test was performed. 87.88% of areas dispensing over the median rate of dispensing were purely rural; however, it is also true that 96.88% of those areas that dispensed below the median range were also rural. With most counties listed as rural, it is hard to correlate the significance of our variables within the group, but if we look at the three highest dispensing rates (233.8, 119.1, 108.1), all were in rural-based areas. The risk of opioid usage, therefore, can be deemed higher in a rural area.

We also looked at the availability of rehabilitation against substance use disorders by geographic location of a hospital. Of the 65 federally qualified healthcare facilities, four offered rehabilitation services against substance use, and 75% were in a non-rural area (N=3). South Dakota has a total area of 75,885 square miles, and its population is expected to be 895,376 in 2021, with 49.8% (N=445,958) of them living in rural South Dakota. In South Dakota, 9.0% of people lack medical insurance [14–18]. The mean per capita income in South Dakota in 2020 was $48,021, and in rural areas it was $40,315, according to the US Developmental Agency (USDA) Economic Research Service (ERS). According to 2020 ACS data, the ERS says that the poverty rate in rural South Dakota is 14.5%, contrasted to 8.6% in the state’s urban districts. We also profiled the top 10 highest dispensers of opioids nationwide based off their population in rural areas (Table 2). We noticed that as the amount of opioid dispensed went down so did the population% age of people living in rural areas.

Aside facing backwardness in rehabilitation and economic aspects, people living in rural areas have lesser access to healthcare facilities, in general, which tampers with the probability of accessing preventative healthcare measures. The state’s inhabitants are in risk of not obtaining the routine, preventative care they need to live healthier lives due to a severe primary care physician shortage in rural South Dakota [4, 19–21]. Part of the reason for the scarcity is a dearth of post-graduate residency opportunities that prepare doctors to practice in rural areas. According to DOHSD, only people who live along the Interstate 29 corridor, a small portion of the middle Missouri River region, and the Black Hills in the west have easy access to primary care providers [12–25]. Furthermore, approximately 1,700 registered nurses departed the South Dakota health sector between 2015 and 2016. At the height of the COVID-19 pandemic in 2020, almost 2,500 nurses left the state service. By the end of this decade, South Dakota will have 2,000 fewer nurses than it needs.

The problem with access to healthcare personnel is heavily worsened by the lack of training sites and isolation engraved by rural areas. 56% of family doctors in practice, according to a research by Fagan et al. [26], worked not just in the same state as their Graduate Medical Education (GME) location but also within a 100-mile radius of it; 29% of participants stayed within a 25-mile radius of the GME training site, and 19% settled within a five-mile radius. In South Dakota, there are now more residency opportunities accessible to recent medical school graduates, though they were not added as rapidly. Three first-year residency positions in the specialty, which specializes in soft-tissue treatments like appendix removals, established in 2014 with the establishment of a new general surgery training program centered in Sioux Falls [27]. At a GME location in Sioux Falls, six additional pediatric residency opportunities have been made available. Another GME location in Sioux Falls announced four new first-year psychiatry resident spots; pathology has gained a new residency place, and three new internal medicine residency positions have also been added [27]. They are all based in Sioux Falls, however, which is a city with no rural areas. Aside not having as many training options, we may look at a rural region like Central South Dakota, we can start to notice why many physicians and providers may further have a problem with finding suitable employment in the state. The next town with above 10,000 residents is more than 100 miles from Pierre in any direction [4]. With prevention, treatment, and rehabilitation out of a feasible plan, patients may have less awareness of complications caused by opioids.

The miscellaneous trends in our statistical venture further embolden rurality as a key player in leading to opioid poisoning. Our bivariate analysis of our secondary database indicates that a patient aged between 10 to 19 years was approximately 50% more likely to visit an ED with an opioid poisoning than someone who was in the next age bracket of 20–29. According to the National Vital Statistics Reports by the Center for Disease Control (CDC), For women of all races and backgrounds, South Dakota had the highest total fertility rate (TFR) in the nation (2,227.5), closely followed by Utah (2,120.5). With a TFR of 1,421.0, Washington, D.C. had the lowest fertility rate in the nation, falling by 57% below South Dakota’s [28–30]. It, therefore, is not surprising for us to see that 18% of the entire population of the state is made up of residents between ages 5 and 18. The state, therefore, has a vital cohort of the most endangered group to opioid poisoning without access to care relating to it.

### Limitations

While this study, to our knowledge, is one of the first to directly correlate the plausibility of an OP to the HRSA-designated rurality status, there could be a few limitations to this study. Firstly, our focus was too vast: because there could be several different codes related to opium-led poisoning, we may have ignored the most frequently coded event at the brink of focusing on the rurality status; this, in turn, may have occluded important finding about the phenomenon the population mostly suffers with. Furthermore, while the data dispensed by DOHSD is a verified tool, it did not specify the rurality of the county. Some places in South Dakota are Native American territories who do not have a direct MUA designation. We had to use census data to ballpark the designation of such territories. The MUA Locator is made by the HRSA, which is a government agency that is responsible to fund medically underserved areas. An agency of the U.S. Department of Health and Human Services; it acknowledges that its definition of rurality is different than that of the census. According to the agency, “The Census overcounts the number of people in rural areas, while the OMB undercounts them” [31]. While this may be true from their analysis, the judgement could present with subjection and bias.

## Conclusions

The opioid crisis is an ongoing conflict within the healthcare sector of the United States with two of three deaths due to drugs is opioid related. Rurality did correlate to the probability of a patient encountering an ED visit due to opioid poisoning. Addiction researchers should focus on key behavioral aspects of patients in rural settings, and government should fund for more preventative and rehabilitative measures, i.e., increasing more rural track GME pathways and psychiatry services in rural hospitals, against substance use. Further studies can correlate rurality to other associated factors to opioid addiction across similar data on a national scale that may bring attention to rural health and maximize quality care for the medically underserved, leading to mitigation of the ongoing opioid crisis.

## Figures and Tables

**Table 1:** HRSA Rural Population-Based Profile by Opioid Dispensing per 100 Showing 9 of 10 States with Majority Rural Counties.

State	Opioid Weights per Person (mg)	% Rural Counties
Alaska	39.62	97%
Montana	35.64	96%
Colorado	33.48	64%
North Dakota	32.19	93%
Wyoming	29.66	86%
South Dakota	25.69	95%
New Mexico	25.33	90%
Oklahoma	25.12	73%
DC	24.39	0%
Nevada	22.95	60%

**Table 2:** Contingency Table of All Counties of South Dakota Based of Rurality and Opioid Dispensing per 100.

variable	NR	PR	R	Total
Yes	3	1	29	33
	9.09%	3.03%	87.88%	50.77%
	100.00%	50.00%	48.33%	
	4.62%	1.54%	44.62%	
No	0	1	31	32
	0.00%	3.12%	96.88%	49.23%
	0.00%	50.00%	51.67%	
	0.00%	1.54%	47.69%	
**Total**	3	2	60	65
	4.62%	3.08%	92.31%	100%
